# Cangma Huadu granules attenuate H1N1 virus-induced severe lung injury correlated with repressed apoptosis and altered gut microbiome

**DOI:** 10.3389/fmicb.2022.947112

**Published:** 2022-08-08

**Authors:** Mingjiang Liu, Tengwen Liu, Xuerui Wang, Chenglong Yu, Tao Qin, Jingui Li, Mina Zhang, Zhenxuan Li, Xuran Cui, Xiaolong Xu, Qingquan Liu

**Affiliations:** ^1^College of Veterinary Medicine, Yangzhou University, Yangzhou, China; ^2^Jiangsu Co-innovation Center for Prevention and Control of Important Animal Infectious Diseases and Zoonoses, Yangzhou, China; ^3^Chengdu University of Traditional Chinese Medicine, Basic Medical College, Chengdu, China; ^4^Beijing Hospital of Traditional Chinese Medicine, Capital Medical University, Beijing, China; ^5^Beijing Key Laboratory of Basic Research With Traditional Chinese Medicine on Infectious Diseases, Beijing, China; ^6^Beijing Institute of Chinese Medicine, Beijing, China

**Keywords:** influenza A virus, gut microbiota, apoptosis, anti-influenza effect, traditional Chinese medicine, Cangma Huadu

## Abstract

Severe influenza A virus infection leads to overwhelming inflammatory responses and cellular apoptosis, which causes lung injury and contributes to high mortality and morbidity. The gut microbiome altered in response to the infection might influence the disease progression and the treatment outcome. Cangma Huadu (CMHD) granules, an in-hospital preparation of traditional Chinese medicine, have been shown to be favorable in the clinical treatment of influenza. However, the effects and mechanisms of CMHD granules on severe influenza pneumonia and its mechanisms are not well-known. In this study, a lethal influenza A (H1N1) A/Puerto Rico/8/34 virus (PR8)-infected mice model was established, and the 16S ribosomal RNA (16S rRNA) V3–V4 region sequencing of the intestinal microbiome was conducted. We revealed that the oral administration of CMHD granules protects mice against higher mortality, enhanced weight loss, overwhelmed interferon-γ concentration, lung viral titers, and severe lung pathological injury in PR8-infected mice. CMHD granules’ administration downregulated the levels of interleukin (IL)-1β, tumor necrosis factor-α, and malondialdehyde, while it upregulated the levels of IL-10, superoxide dismutase, and glutathione peroxidase. Subsequently, it decreased the protein ratio of B-cell lymphoma-2/Bcl-2-associated X and the expression of cleaved caspase-3. The diversity and compositions of the gut microbes were altered profoundly after the administration of CMHD granules in PR8-infected mice. A higher abundance of *Bifidobacterium*, *Parasutterella*, *Bacteroides*, and *Faecalibaculum* was observed in the CMHD group, and a higher abundance of *Lactobacillus* and *Turicibacter* was observed in the positive drug Ribavirin group. The linear discriminant analysis effect size also revealed a higher proportion of *Bacteroides* and *Bifidobacterium_pseudolongum* characterized in the CMHD group. These results demonstrated that CMHD granules are a promising strategy for managing severe influenza and attenuating severe lung damage *via* reducing viral titer, inflammatory responses, and oxidative stress. The mechanisms are involved in repressed Bcl-2-regulated apoptosis and altered composition and diversity of the gut microbiome.

## Introduction

Over the past decades, RNA viruses have caused huge global impacts on economic chaos, global public health resources, and, most importantly, human health. Influenza is an acute contagious viral infection with both seasonal and pandemic modes in humans (Bridges et al., 2003; Morens and Taubenberger, 2019). It is reported that seasonal influenza affects up to 10% of the adult population and 20% of children annually and shows a substantial incidence (Peteranderl et al., 2016). The continuing challenges of the influenza A virus (IAV) pandemic include the emergence of the H1N1 pandemic in 2009, human infection with H7N9 avian influenza in 2013, and the sporadic infection of highly pathogenic H5N1 avian influenza. The IAV can affect organs and manifests as an acute febrile illness with variable degrees of systemic and respiratory symptoms (Krammer et al., 2018). Considering the disease progression of IAV infection, two outcomes should be given due attention. First, IAV enters host cells and causes acute lung injury, leading to hospitalization. Second, uncontrolled lung injury leads to secondary bacterial pneumonia, which turns the patient’s condition into a severe situation (Klomp et al., 2021). Although the IAV is one of the most deeply studied pathogens, the existing control schemes need further improvement. Influenza vaccines must be updated regularly because of the continuous antigen drift and sporadic antigen transfer in the virus surface glycoprotein. At present, IAV infection treatment is limited to neuraminidase inhibitors; therefore, there is an urgent need for new drugs and vaccine methods.

In terms of mechanism, infected epithelial cells started apoptosis and necroptosis, representing the primary form of damage to the epithelium during the early stages of IAV infection. Damaged cells in the apoptotic cascade promote the activation of caspases and further trigger immune and inflammatory response (Chung et al., 2006). Moreover, virus-infected epithelial cells release chemokines, which recruit a large number of myeloid and lymphoid cells to the lung tissue. The excessive inflammatory response in IAV-infected lungs can lead to acute respiratory distress syndrome, leading to severe lung injury and respiratory failure (Kalil and Thomas, 2019; Stegelmeier et al., 2019). Thus, modulating the apoptosis and inflammatory response can prevent the occurrence and development of lung injury. Traditional Chinese medicine (TCM) has been reported to elicit anti-inflammatory and anti-apoptosis effects in those who underwent diseases. Our previous studies reported that herbal treatment altered microglial polarization from M1 phenotype to M2 phenotype to reduce the pro-inflammatory cytokine production (Wang et al., 2020) and prevented apoptosis by suppressing endoplasmic reticulum- and mitochondria-associated pathways in animal models of lethal infection (Xu et al., 2018). The mechanisms of TCM in the treatment of viral infection have also been proved to be related to the reduction of cytokine storm caused by hyper-release of interferon (IFN), interleukins (IL), tumor-necrosis factors (TNF), chemokines, and cytokines (Huang et al., 2021).

The microbiota plays an essential role in the host disequilibrium transition from homeostasis to disease. Disrupted intestinal bacterial composition is associated with various diseases and dysfunctions, including autoimmunity, inflammatory disorders, and infectious diseases (Cho and Blaser, 2012). Accumulating evidence showed that balanced gut microbiota plays a protective role in the body against infections such as IAV (Stefan et al., 2020), *Klebsiella pneumonia* (Sequeira et al., 2020), *Staphylococcus aureus* (Hu et al., 2020), and *Streptococcus pneumonia* (Schuijt et al., 2016). The natural resistant mechanisms of the gut microbiota after IAV infection have been proved to be related to type I interferon signaling pathways (Steed et al., 2017; Bradley et al., 2019) and the activation of the inflammasome and T lymphocytes (Ichinohe et al., 2011). Gut commensal microbes are essential for the decomposition of nutritional products and critical producers of essential metabolites (Valdes et al., 2018). As one of the natural characteristics of TCM, only partial components can be absorbed directly into the blood; the secondary digestion and decomposition of the active ingredients by gut microbiota are indispensable (Wang et al., 2021a). TCM nutraceuticals, such as indigestive carbohydrates/polysaccharides, polyphenols, alkaloids, could pass through the stomach and reach the intestine and are frequently fermented or converted by the local gut microbiota to form bioactive and bioavailable metabolites (Lin et al., 2021). Therefore, exploring the interaction between TCM components and gut microbiota may better clarify its mechanisms under diseases.

Historically, TCM has worked well in treating infectious diseases (Huang et al., 2021). During the IAV pandemic in 2009, four anti-flu TCM prescriptions with outstanding clinical efficacy were recommended by the Chinese government to treat H1N1 influenza (Ge et al., 2010; Li et al., 2016). Cangma Huadu (CMHD) granules are a new TCM prescription approved in 2020 by the Beijing Food and Drug Administration (Z20200008000) for the clinical treatment of RNA virus infections, including influenza and SARS-CoV-2. We previously reported that CMHD granules protect mice from mild IAV infection (Cui et al., 2022). However, the underlying mechanism and molecular effects of CMHD granules on lethal influenza remain unclear. In this study, we established an animal model of IAV-induced lethal pneumonia, and the protective effects of CMHD granules on severe lung injury and excessive inflammatory response were investigated. Moreover, we assessed the regulation of CMHD granules on the microbiota of IAV-infected mice. Our findings provide evidence to support the use of CMHD granules for the treatment of IAV infection and gain insights into its underlying regulatory role in the host and microbe.

## Materials and methods

### Animals

Male ICR mice (18–22 g) were obtained from the Comparative Medicine Center of Yangzhou University [Jiangsu, China Certificate Number: SYXK(E)2017-0044]. All animals were housed under specific pathogen-free conditions (23 ± 1°C; 50 ± 10% humidity, 12 h light/dark cycle) with free access to standard food and water. All animal experiments were conducted under the Animal Care and Use Committee of Yangzhou University. All experimental animal ethics are approved by the Experimental Animal Ethics Committee of Yangzhou University (No. 202103-009).

### Influenza A virus infection

The mouse-adapted A/Puerto Rico/8/34(H1N1) influenza virus strain (referred to as PR8) was preserved in the College of veterinary medicine, Yangzhou University, China. Mice were anesthetized with pentobarbital sodium (40 mg/kg) by intraperitoneal injection. When sufficient depth of anesthesia was confirmed and breathing occurred at 2–3-s intervals, mice were intranasally inoculated with 50 μL 10^4^.^4^ EID50 PR8 virus.

### Preparation of Cangma Huadu granules

Cangma Huadu granules contain 8 Chinese herbs, including 30 g Cangzhu (*Rhizoma atractylodis*) (30/106), 10 g Mahuang (*Herba ephedra*) (10/106), 10 g Huoxiang (*Herba Agastache*) (10/106), 15 g Shegan (*Rhizoma belamcandae*) (15/106), 10 g Shandougen (*Radix sophora tonkinensis*) (10/106), 10 g Ercha (*Acacia catechu*) (10/106), 15 g Jindenglong [*Physalisalkengi L.var.franchetii (Mast.) Makino*] (15/106), and 6 g Shenggancao (*Glycyrrhiza uralensis Fisch*) (6/106). All the herbs were provided by the pharmacy of Beijing Hospital of Traditional Chinese Medicine, China. A total of 106 g of crude drugs were soaked and decocted using distilled water for 30 min and then concentrated to a low dosage of 0.5 g/mL (CMHD-L groups), a medium dosage of 1 g/mL (CMHD-M groups), and a high dosage of 2 g/mL (CMHD-H groups) as final dosages.

### Treatment protocols

The mice were randomized and divided into six groups as follows: the control (Con), the model (Mod), the CMHD low dosage (CMHD-L), the CMHD medium dosage (CMHD-M), the CMHD high dosage (CMHD-L), and the Ribavirin group (RBV). At 1 day post-infection (dpi), CMHD-L, CMHD-M, and CMHD-H groups were orally administrated with 0.2 mL decoction of 0.5, 1, and 2 g/mL CMHD granules, respectively, one time daily for seven continuous days. The mice in the RBV group were orally administrated with 0.2 mL of 0.075 g/kg Ribavirin (Zhejiang Chengyi Pharmaceutical Co., Ltd, Wenzhou, China) that dissolved in dd H2O, one time daily from 1 dpi for seven continuous days. Mice in the Con and Mod groups were orally administrated with 0.2 mL of dd H_2_O one time daily from 1 dpi for seven continuous days. The weight of mice in each group was monitored every day for 7 days. On 7 dpi, lung tissue and fecal samples of mice were collected for weighing, pathology, and 16S rRNA sequencing, respectively.

### Lung index

Lungs were directly excised surgically and weighed. The lung index is the ratio of organ/body weight and was calculated according to the following formula: Lung index = (Lung weight/Body weight) × 100%.

### Histopathological examination

For histopathological analysis, the lung tissues of mice were harvested, fixed in 4% formaldehyde for 24 h, embedded in paraffin, and then sliced into 4 μm-thick sections with a slicer (LEICA RM2255, Germany). The sections were stained with hematoxylin and eosin. An Olympus microscope (BX53F, Tokyo, Japan) was used for histological observation.

### Lung viral titration

Lung viral titration was evaluated by the PR8 mRNA expression of Real-time quantitative polymerase chain reaction (Real-time PCR). Total RNA was extracted using Trizol (Vazyme, China) reagent according to the instructions. Its quantity was detected by the nanodrop spectrophotometer (Thermo Scientific, Wilmington, DE, United States), and cDNA was synthesized using Hiscript II QRT supermax (Vazyme, China). Finally, the real-time PCR was performed using a CFX96™ connected real-time PCR system (Bio-Rad, United States) with SYBR Green PCR Master Mix (Vazyme, China). The cycle conditions were as follows: an initial denaturation step at 95°C for 30 s, 40 cycles of denaturation for 5 s at 95°C, and annealing and extension for 30 s at 60°C. The quantification results were calculated using the 2−ΔΔCT method compared to GAPDH as the reference mRNA. Primers for real-time PCR were from Tsingke Biotechnology, China. Their sequences were listed below as follows: PR8: F-3′ TAGCATGCATGCTATCGGTACGT5′, R-3′TAGCTATCTAGC TAGCTAGCTA5′, and GAPDH: F-3′GGTGAAGGTCGGTG TGAACG5′, R-3′CTCGCTCCTGGAAGATGGTG5′.

### Inflammatory cytokines and oxidative stress measurement

Mouse ELISA kits for IL-1β (RK00006), IL-10 (RK00006), TNF-α (RK00027), and IFN-γ (RK00019) were all purchased from ABclonal Biotechnology Co., Ltd., China. The oxidative stress kits of superoxide dismutase (SOD), glutathione peroxidase (GSH-Px), and malondialdehyde (MDA) were all purchased from Nanjing Jiancheng Bioengineering Institute, China. All measurements were carried out according to the manufacturer’s instructions.

### Western blotting analysis

The primary antibodies against B-cell lymphoma-2 (Bcl-2) (3498), Bcl-2-associated X (Bax) (2772), cleaved-caspase-3 (Asp175) (9661), and β-actin (4970) were all from Cell Signaling Technology (Danvers, MA, United States). Total protein was extracted from the left lower lobe using the ice-cold RIPA buffer (APPLYGEN). After protein was quantified with a BCA protein assay kit (Beyotime Institute of Biotechnology, China) and boiled with loading buffer (Solarbio, China), equal amounts of protein in each sample (100 μg) were prepared for electrophoresis on 8–12% SDS-PAGE and then electro-blotted onto PVDF membranes (Merck Millipore, United States). The membrane was incubated with 5% skim milk for 1 h at room temperature (RT), followed by incubation overnight with primary antibodies at 4°C (all antibodies were diluted following instructions). After the primary antibody was incubated, it was washed three times with TBST and then incubated with species-specific horseradish peroxidase-conjugated secondary antibodies (diluted 1:5,000) at RT for 1 h. After three washes with TBST, protein signal bands were visualized on a Chemidoc XRS (Bio-Rad, Marnes-la-Coquette, France) by an enhanced Chemiluminescence kit. Densitometry quantification of immunoblot signals was performed using ImageJ software. Each experiment was performed in triplicate.

### DNA extraction and high-throughput 16S rRNA sequencing

Fresh fecal pellets were collected, stored in liquid nitrogen, and then immediately stored in a refrigerator at −80°C before extraction of total DNA. The 16S rRNA gene comprising V3–V4 regions was amplified using the standard primer pair and quality-controlled using the common primer pair and the microbial diversity analysis. Bacteria primers: 341F, CTAYGGGBRGCASCAG; 806R, GGACTACNNGGGTATCTAAT. Fungal ITS1-2 regions were sample barcodes and sequencing adaptors. Fungal primers: ITS1-1F-F, CTTGGTCATTTAGAGGAAGTAA; ITS1-1F-R, GCTGCGTTCTTCATCGATGC. Briefly, the raw sequences were quality-controlled using QIIME with default parameters and then demultiplexed and clustered into species-level (97% similarity) operational taxonomic units (OTUs). Species annotation analysis was performed using the Mothur method and SILVA138’s SSUrRNA database (set threshold 0.8∼1). α-Diversity and β-diversity analyses were performed using QIIME. Discriminative taxa were determined using LEfSe (LDA Effect Size). The 16S rRNA gene sequence of the entire prokaryote genome was extracted from the KEGG database and compared with the SILVA SSU Ref NR database (BLAST bitscore > 1500). The correlation matrix was established and the whole genome functional information of prokaryotes annotated by UProc and PAUDA in the KEGG database corresponded to the SILVA databases so that OTU clustering of functional annotation sequencing samples in SSU was achieved.

### Statistical analyses

The experimental data were analyzed using SPSS 25.0 statistic software. Diagrams were performed by using GraphPad Prism 8.0 software. The results were compared using the one-way analysis of variance (ANOVA). The differences were considered significant at a *P*-value < 0.05. All the experimental data were presented as the mean ± SEM.

## Results

### Cangma Huadu granules reduced the mortality of influenza A virus infection

**FIGURE 1:**
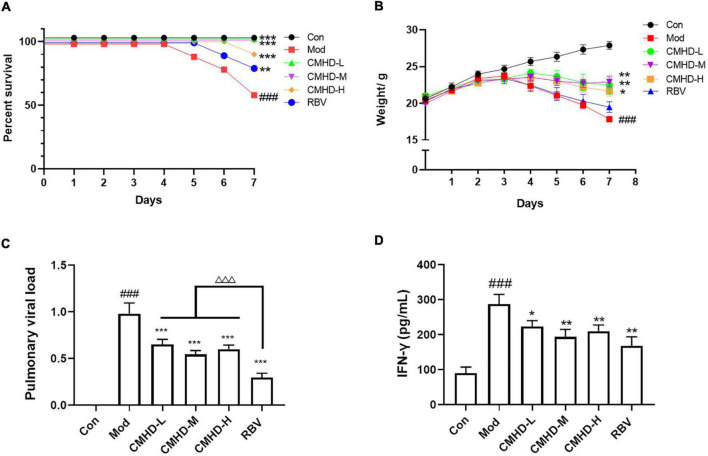
CMHD granules attenuated weight loss and virus titer after IAV infection. ICR mice were infected with 50 μL 10^4^.^4^ EID50 influenza A/Puerto Rico/8/1934 H1N1 (PR8) virus following treatment with dd H2O (0.2 ml, oral administration) (Mod), CMHD granule (0.5 g/ml, 0.2 ml, oral administration) (CMHD-L), CMHD granule (1 g/ml, 0.2 ml, oral administration) (CMHD-M), CMHD granule (2 g/ml, 0.2 ml, oral administration) (CMHD-H), or Ribavirin (0.2 Ml, 0.075 g/kg, oral administration) (RBV) per day for seven consecutive days. Mice in the control group (Con) were not infected with a virus and perfused with dd H2O (0.2 ml, oral administration) per day for even consecutive days. **(A)** Kaplan–Meier curves for 7 days. **(B)** Body weight for 7 days. **(C)** Comparison of pulmonary viral load among different groups at 7 dpi. **(D)** Comparison of interferon–γ content in lung tissues among different groups at 7 dpi. Values are mean ± SEM of 10 animals per group. ^#^Compared with Con, *compared with Mod, ^△^compared with RBV. *p < 0.05, **p < 0.01, ***p < 0.001 of each symbol, respectively.

To verify the effects of CMHD granules on the mortality of lethal influenza, a nasal drip of a 50 μL 10^4^.^4^ EID50 mouse-adapted influenza A/Puerto Rico/8/1934 H1N1 (PR8) lethal dose virus was used to establish a pneumonia model, and the mortality of animals in each group was observed for 7 days. As seen in Figure 1A, all sham animals survived throughout the 7 days. Compared with the control group, the mortality of mice in the model group was significantly higher (7-day mortality 0 vs. 40%, median death time > 7 days vs. 6.25 ± 0.92 days). Compared with the model group, low, medium, and high doses of CMHD granules significantly reduced the mortality of infected mice and prolonged the median death time to 7 days or more. As a positive drug control, Ribavirin significantly reduced the mortality of infected mice compared with the model group. There were no significant differences in mortality and median time of death between CMHD granules and Ribavirin administration.

### Cangma Huadu granules attenuated weight loss and virus titer after influenza A virus infection

To further verify the therapeutic effect of CMHD granules on lethal influenza pneumonia, the body weight and viral load of mice in each group were measured. As seen in Figure 1B, the weight of the control group mice increased slowly from 0 to 7 dpi. Compared with the control group, the weight in the model group turned and began to decrease at 3 dpi. Compared with the model group, the low, medium, and high doses of CMHD granules groups showed less weight loss. The body weight of the model group decreased significantly compared with the control group. The weight loss was significantly improved after the administration of low, medium, and high doses of CMHD granules compared to the model group. Ribavirin showed no effect on attenuating weight loss after IAV infection.

As seen in Figure 1C, the pulmonary viral load in the model group was significantly higher than that of the control group at 7 dpi. The viral load was significantly decreased after low, medium, and high doses of CMHD granules administration compared to the model group. Ribavirin showed the best effect on reducing the viral load among all the groups. The levels of IFN-γ, a key restriction component during IAV replication and transmission, were also detected (Figure 1D). The content of IFN-γ in the lung tissue of the model group increased significantly compared with the control group. Low, medium, and high doses of CMHD granules administration showed a similar effect as Ribavirin on decreasing the level of IFN-γ.

### Cangma Huadu granules alleviated severe lung damage during influenza A virus airway infection

**FIGURE 2:**
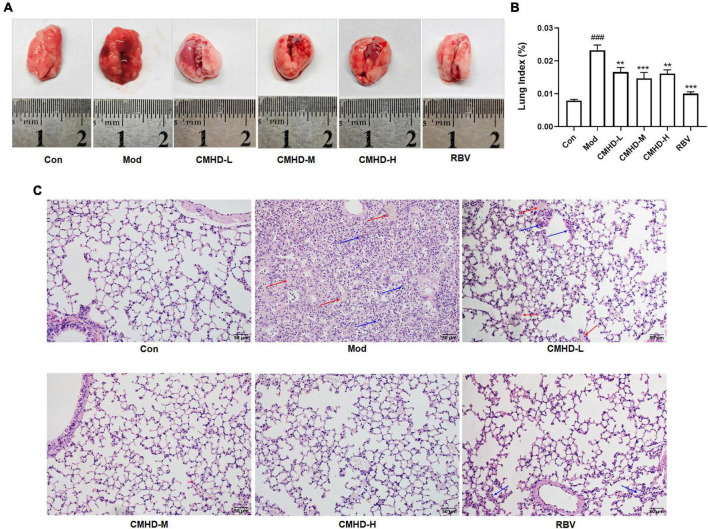
CMHD granules alleviated severe lung damage during IAV airway infection. **(A)** The general morphology of lung tissue and **(B)** lung index in uninfected or infected mice following the administration of CMHD granules or Ribavirin at 7 dpi. **(C)** Histopathological injury of representative lung sections in each group at 7 dpi with 400 × magnification (Bar = 50 μm). The blue arrow indicates inflammatory cell infiltration and the red arrow indicates erythrocyte exudation. Values are mean ± SEM of 10 animals per group. ^#^Compared with Con, ^∗^compared with Mod. ^∗∗^*p* < 0.01, ^∗∗∗^*p* < 0.001 of each symbol, respectively.

We further evaluated the effect of CMHD granules on lung damage after IAV infection. As the general morphology of lungs displayed in Figure 2A, significant changes, including larger volume, increased edema, and bleeding, were observed in mice after IAV infection compared to the control. Low, medium, and high doses of CMHD granules or Ribavirin administration significantly improved the lung injury after infection, including reduced volume, edema, and bleeding. The alternation of lung index was consistent with the trends of the general morphology (Figure 2B). Compared with the control group, the lung index in the model group was significantly higher. Different doses of CMHD granules or Ribavirin administration significantly reduced the lung index caused by infection. HE staining showed that the pathological morphology of lung tissue in the control group was normal. Compared with the control group, the alveolar structure of the model group was incomplete. Cell necrosis, expansion of airspaces, and infiltration of the inflammatory cells were displayed. After administration with different doses of CMHD granules or Ribavirin, the destruction of the alveolar structure was attenuated, with less necrosis and inflammatory cell infiltration than the model group (Figure 2C).

### Cangma Huadu granules regulated the inflammatory cytokine secretion and oxidative stress upon influenza A virus infection

**FIGURE 3:**
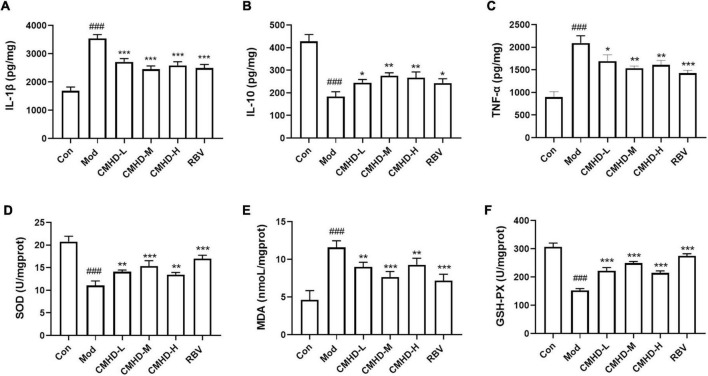
CMHD Granules regulated the inflammatory cytokine secretion and oxidative stress upon IAV infection. Lung tissue levels of **(A)** Interleukin-1β (IL-1β), **(B)** interleukin-10 (IL-10), **(C)** tumor necrosis factor-α (TNF-α), **(D)** superoxide dismutase (SOD) activity, **(E)** malondialdehyde (MDA), and **(F)** glutathione peroxidase reactive (GSH-Px) activity detected from uninfected or infected mice following the administration of CMHD granules or Ribavirin at 7 dpi. Values are mean ± SEM of 10 animals per group. ^#^Compared with Con,*compared with Mod. *p < 0.05, **p < 0.01, ***p < 0.001 of each symbol, respectively.

To better explore the mechanism of CMHD granules in reducing the mortality of infected mice, we detected the secretion of inflammatory cytokines and the alteration of oxidative stress in the lung tissue at 7 dpi. As seen in Figures 3A–C, IAV-infected mice demonstrated considerably higher levels of IL-1β and TNF-α and reduced levels of IL-10 compared to the control group. Different dosages of CMHD granules or Ribavirin administration showed similar effects in reducing the levels of IL-1β and TNF-α and increasing the level of IL-10 in lung tissue compared to the model group.

As seen in Figures 3D,F, the SOD and GSH-Px activities of the lung tissue in the model group were significantly higher than that in the control group. The administration of CMHD granules or Ribavirin significantly improved the upregulation of SOD and GSH-Px activities caused by infection. As shown in Figure 3E, the MDA level in the lung tissue of the model group was significantly lower than that in the control group. The administration of CMHD granules or Ribavirin significantly reduced the MDA level compared to the model group. Therefore, CMHD granules have specific anti-inflammatory and anti-oxidative effects under IAV infection.

### Effects of Cangma Huadu granules on the Bcl-2/Bax/caspase-3 signaling pathway in influenza A virus-infected mice

**FIGURE 4:**
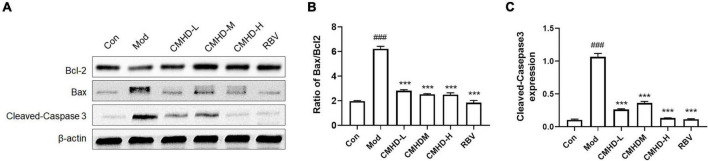
Effects of CMHD granules on the Bcl-2/Bax/caspase-3 signaling pathway in IAV-infected mice. **(A)** Representative Western Blots of B-cell lymphoma-2 (Bcl-2) family, including Bcl-2 and Bcl-2-associated X (Bax), and cleaved caspase-3 in lung tissues of uninfected or infected mice following the administration of CMHD granules or Ribavirin at 7 dpi. The same membranes were probed with β-actin. **(B)** Densitometric analysis of Bax/Bcl-2 ratio on protein levels, **(C)** and the expression of cleaved caspase-3. Values are mean ± SEM of 10 animals in each group. ^#^Compared with Con, *compared with Mod. ***p < 0.001 of each symbol, respectively.

To further explore the mechanism of CMHD granule on lethal IAV infection, we detected the protein levels of the Bcl-2 family, including Bcl-2 and Bax, and the level of cleaved-caspase-3, which are related to the regulation of apoptosis in lung tissues (Figure 4A). Compared with the control, the relative protein expression of cleaved-caspase-3 and the ratio of Bax/Bcl-2 were significantly increased in the lung tissue of infected mice (Figure 4B,C). Compared with the model group, low, medium, and high doses of CMHD granule or Ribavirin administration significantly downregulated the protein expression of cleaved-caspase-3 and Bax/Bcl-2 ratio. These results revealed that CMHD administration might inhibit apoptosis after IAV infection in a manner associated with the regulation of the Bcl-2/Bax/caspase-3 signaling pathway.

### Cangma Huadu granules altered gut microbial structural diversity

**FIGURE 5:**
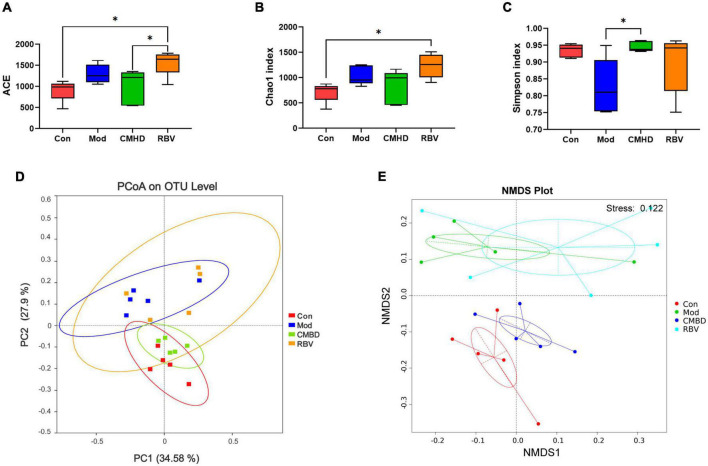
CMHD granules altered fecal microbial diversity in IAV-infected mice. 16S rRNA sequencing of the fecal samples in the control (Con), the model (Mod), the CMHD granules administration (CMHD), and the Ribavirin administration groups (RBV) were detected at 8 dpi (5 animals per group). α-Diversity was evaluated based on the Ace **(A)**, Chao1 **(B)**, and Simpson **(C)** indices of the operational taxonomic unit (OTU) levels. ^∗^*P* < 0.05. β-Diversity was evaluated based on the principal coordinate analysis (PCoA) **(D)** and non-metric multi-dimensional scaling (NMDS) analysis **(E)** of the OTU levels.

The α-diversity, including Ace, Chao1, and Shannon, indexes were evaluated to determine the ecological diversity within a microbial community after CMHD granules or Ribavirin administration in IAV-infected mice. As seen in Figures 5A–C, ACE and Chao1 indexes in the model group tended to increase, but there was no significant difference in Ace, Chao1, and Shannon indexes of the model group compared with the control group. Ace and Chao1 indexes in the Ribavirin group were significantly higher than those in the control group. Compared with the model group, the Shannon index of the CMHD group was significantly higher. The ACE index in the Ribavirin group was higher than that in the CMHD group. The β-diversity was measured by the principal coordinate analysis (PCoA) (Figure 5D) and the non-metric multidimensional scaling (NMDS) analysis (Figure 5E). PCoA based on the Bray–Curtis dissimilarity index showed a significant difference among the control, the model, the CMHD, and the Ribavirin groups (ANOSIM *P* < 0.001). NMDS analysis intuitively reflected the difference in distance within and among the groups. The control group and the CMHD group were distributed below NMDS2 0.0, while the model group and the Ribavirin group were distributed above NMDSs2 0.0. The NMDA Stress is 0.12 (less than 0.2), which indicates that the model is reliable. Overall, CMHD granules or Ribavirin administration significantly altered the structural microbial diversity of the intestine in IAV-infected mice.

### Cangma Huadu granules altered the dominant bacteria after influenza A virus infection

**FIGURE 6:**
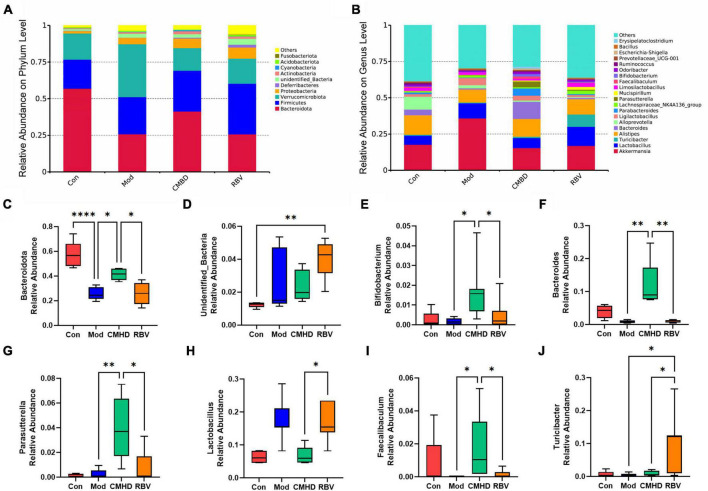
CMHD granules altered the dominant bacteria and microbiota composition in IAV-infected mice. The percentage of community abundance at the phylum **(A)** and genus **(B)** levels in the control (Con), the model (Mod), the CMHD granules administration (CMHD), and the Ribavirin administration groups (RBV). Data were shown as relative abundance (%) of the top 10 abundant phylum and top 20 abundant genera in each group. **(C–J)** Significant different bacterium among the Con, Mod, CMHD, and RBV groups. Statistical analysis was performed using the one-way ANOVA. ^∗^*P* < 0.05, ^∗∗^*P* < 0.01, ^∗∗∗∗^*P* < 0.0001.

As seen in Figure 6A, Bacteroidota, Firmicutes, Verrucomicrobiota, Proteobacteria, and unidentified Bacteria were the dominant phyla microbiota (relative abundance > 1%) in the four groups. In addition, Actinobacteria was a dominant phyla microbiota in the model, the CMHD, and the Ribavirin groups. Deferribacteres was a dominant phyla microbiota in the Ribavirin group. The average abundance of Bacteroidota and unidentified Bacteria showed significant differences among the four groups using one-way ANOVA.

As seen in Figure 6B, the top 3 predominant genera were *Akkermansia* (17.7%), *Alistipes* (13.4%), and *Alloprevotella* (8.8%) in the control group; *Akkermansia* (35.9%), *Lactobacillus* (10.4%), and *Alistipes* (9.0%) in the model group; *Akkermansia* (15.4%), *Alistipes* (12.3%), and *Bacteroides* (11.7%) in the CMHD group; and *Akkermansia* (16.9%), *Lactobacillus* (13.2%), and *Alistipes* (10.9%) in the Ribavirin group, respectively. The average abundance of *Lactobacillus*, *Parasutterella*, *Turicibacter*, *Bacteroides*, *Faecalibaculum*, and *Bifidobacterium* showed significant differences among the four groups using one-way ANOVA.

### Cangma Huadu granules altered fecal microflora composition after influenza A virus infection

As seen in Figure 6C, in the phylum, the abundance of Bacteroidota decreased in the model group compared with the control group, increased in the CMHD group compared with the model group, and decreased in the Ribavirin group compared with the CMHD group, respectively. As seen in Figure 6D, the abundance of unidentified Bacteria increased in the model group compared with the control group. As seen in Figures 6E–J, in the genera, the abundance of bacterium including *Bifidobacterium*, *Parasutterella*, *Bacteroides*, and *Faecalibaculum* was increased in the CMHD group compared with the model group. The abundance of bacterium including *Bifidobacterium*, *Parasutterella*, *Bacteroides*, and *Faecalibaculum* was decreased, while the bacterium *Lactobacillus* and *Turicibacter* were increased in the Ribavirin group compared with the CMHD group.

**FIGURE 7:**
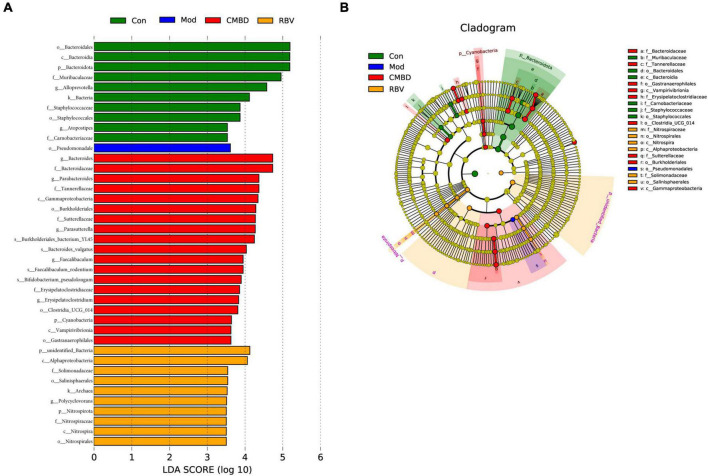
Linear discriminant analysis (LDA) integrated with effect size (LEfSe). **(A)** The differences in abundance among the control (Con), the model (Mod), the CMHD granules administration (CMHD), and the Ribavirin administration groups (RBV). **(B)** Cladogram indicating the phylogenetic distribution of microbiota correlated with each group.

Moreover, LEfSe was used to generate a cladogram to identify the specific bacteria in each group. Using a logarithmic LDA score cutoff of 3.5, we identified 40 bacteria as key discriminants (Figure 7). Higher proportions of bacterium including Bacteroidota, *Alloprevotella*, and *Atopostipes* were identified in the control group, while a higher proportion of *Pseudomonadale* was identified in the model group. A higher proportion of genera including *Paeasutterlla*, *Bacteroides*, and *Bifidobacterium_pseudolongum* were identified in the CMHD group, while a higher proportion of unidentified_Bacteria and *Nitrospirota* were identified in the Ribavirin group.

## Discussion

The development of influenza infection into a severity situation is mainly caused by excessive lung injury and secondary pneumonia (Herold et al., 2015). Two factors are responsible for the exacerbation of the disease and mortality, including lung tissue damage and overwhelming inflammatory response (McCullers and Bartmess, 2003). We previously found that CMHD has therapeutic effects on mild IAV infection by using a non-lethal dose of 35 μL 14LD50 H1N1/FM1 virus infection mouse model (Cui et al., 2022). This study established a lethal influenza model using a dosage of 50 μL 10^4^.^4^ EID50 H1N1/PR8 virus. According to our data, 7-day mortality of the pneumonia model is about 40%, indicating a lethal infection. Treatment with CMHD granules at different doses could decrease the mortality of infected mice. Besides, we noticed that CMHD granules attenuated the weight loss induced by a virus infection, indicating an improvement in the condition. Considering the severity of this model, CMHD granules possess a promising therapeutic effect on preventing severe pneumonia caused by IAV. Anti-viral drug Ribavirin showed similar therapeutic efficacy in the mice with lethal infections. However, based on our understanding, the anti-viral effect of TCM medicine is usually not as effective as designed in western medicine. Therefore, we detected the viral titer in mice of the model group and the treatment group. Not surprisingly, the direct anti-viral property of CMHD granules is inferior to Ribavirin. Taken above, we hypothesize that the curative effect of CMHD granules is associated with its regulatory role in either inflammatory response or lung injury.

Previous studies showed that TCM has anti-inflammatory and antioxidant effects in RNA virus infections. Yang et al. reported that different TCM compounds have inhibitory effects on the gene levels of the pro-inflammatory cytokine, including TNF-α, IL-6, CCL-2, and IFN-γ in coronavirus (SARS-CoV-2) infection (Runfeng et al., 2020; Ma et al., 2021). Our study demonstrated that CMHD granules inhibit the release of pro-inflammatory factors, including TNF-α, IL-1β, and IFN-γ, promote the release of anti-inflammatory factor IL-10, and reduce oxidative stress after IAV infection. The increased levels of inflammatory cytokines and reactive oxygen species after IAV infection activate apoptosis and autophagy *in vivo* (Mehrbod et al., 2019). Thus, the endogenous apoptosis pathways, which are strictly controlled by Bcl-2 gene family of proteins, were also evaluated in this study. We found that the ratio of cell death agonists, Bax, vs. cell death antagonists, Bcl-2, decreased after CMHD and Ribavirin administration, alongside the decreased level of executioner caspases, caspase-3. However, as a previous study pointed out that the gut bacteria are necessary for the initiation and maintenance of tissue repair but are unnecessary for the rapid drug-mediated apoptosis as a chemotherapeutic drug’s mechanisms (Rigby et al., 2016), and whether the regulation of apoptosis and gut bacteria after CMHD administration is in a parallel or causal relationship needs to be verified in the future.

The influence of the gut microbiota on the pathogenicity of the influenza virus is profound as it can not only affect viral infections in the gastrointestinal system but also produce metabolites or influence different lymphocytes to modulate influenza infections in the lung (Ivanov et al., 2009). Studies have proved the protective effects of gut microbiota against respiratory and systemic infection, especially after influenza virus infection (Brown et al., 2017). In our study, it is notable that the shifted microbiota diversity and composition after CMHD administration tended to be similar to the control. In contrast, the alternation after Ribavirin administration was similar to those in the model group, particularly in the NMDA analysis. We found no statistical difference in the α-diversity between infected and uninfected mice, which is consistent with previous studies. However, they have pointed out the reduction of commensal community richness (Yildiz et al., 2018) and the different α-diversity between the IAV infected-death and infected-survival mice (Zhang et al., 2020). Although the ACE index of α-diversity increased after Ribavirin compared with CMHD administration, this is likely due to the increased abundance of unidentified_Bacteria rather than the beneficial commensal community.

We identified a series of genera characteristics of gut microbes that are closely related to CMHD or Ribavirin administration in IAV-infected mice, including *Bifidobacteria*, *Lactobacillus*, *Bacteroides*, *Parasutterella*, *Faecalibaculum*, and *Turicibacter*. *Bifidobacteria* and *Lactobacillus* are common probiotic strains, which have been successfully applied in the food and pharmaceutical industries. Several of them confirmed to have protective effects on IAV infection, including *Bifidobacterium animalis* (Zhang et al., 2020), *Bifidobacterium longum* (Kawahara et al., 2015), and *Lactobacillus rhamnosus* (Villena et al., 2012). The gut population of endogenous *B. animalis* expanded to enhance host influenza resistance during lethal influenza infection. Oral administration of *B. animalis* alone or with *B. pseudolongum* combination significantly increased the survival rate of infected mice (Zhang et al., 2020). Li et al. demonstrated through fecal transplantation experiments that part of the mechanisms of immune regulation of TCM after influenza virus infection is *via* gut microbiota (Deng et al., 2021). Apparently, the significant upregulation of *Bifidobacterium* and *Bifidobacterium_pseudolongum* abundance by CMHD administration was beneficial, which might be one of its mechanisms in treating IAV infection. However, Ribavirin has a better effect on the upregulation of *Lactobacillus* abundance than CMHD. We also found a higher abundance of *Bacteroides*, *Parasutterella*, and *Faecalibaculum* after CMHD administration. *Bacteroides* are beneficial bacteria, which metabolize polysaccharides and oligosaccharides and provide nutrition and vitamins to the host (Zafar and Saier, 2021). A previous study reported preferentially deleted *Bacteroides* in IAV infection mice, which was speculated to be a reason for superinfection sensitivity following IAV infection (Yildiz et al., 2018). There are limited reports on the role of *Parasutterella* and *Faecalibaculum* in influenza infection. However, under other disease conditions, *Parasutterella*, as a succinate producer, played an important role in bile acid maintenance and cholesterol metabolism (Ju et al., 2019), and *Faecalibaculum*, as a short-chain fatty acids producer, reduced tumor growth (Zagato et al., 2020). The effect of Ribavirin on the increase of the bacterial abundance of *Turicibacter* was more significant than that of CMHD. *Turicibacter* is a common genus of Firmicutes, which is closely related to lipid and carbohydrate metabolism. As a potential beneficial genus, studies showed that *Turicibacter* protects the host from chronic and inflammatory diseases (Wang et al., 2021b). Based on these aforementioned findings, we speculated that the mechanism of CMHD granules is related to the alternation of the microbiota structure and some specific microbiota in the intestine.

## Conclusion

Our study provided a novel and effective TCM compound, CMHD granules, for treating lethal influenza. Although the administration of the positive drug Ribavirin showed an excellent effect in reducing viral load, CMHD granules exhibited similar effects on reducing mortality and alleviating lung injury after IAV infection. Our study further underlined the crucial mechanisms of CMHD granules, including reducing virus load, overwhelmed inflammatory responses, oxidative stress, and apoptosis. In addition to these, CMHD granules displayed pronounced beneficial effects on regulating the diversity and composition of gut microbiota after IAV infection. Noteworthy, bacterium protecting hosts against extraintestinal infections, including *Bifidobacterium*, *Bifidobacterium_pseudolongum*, and *Bacteroides*, became more abundant after CMHD granules administration. Nevertheless, Ribavirin showed a better effect on up-regulating the abundance of *Lactobacillus* and some potentially beneficial bacteria, such as *Turicibacter*. In general, this promising example of TCM compound protected hosts against severe influenza, which is comparable to Ribavirin.

## Data availability statement

The data presented in this study are deposited in the SRA database, accession number PRJNA860266.

## Ethics statement

The animal study was reviewed and approved by the Experimental Animal Ethics Committee of Yangzhou University (No. 202103-009).

## Author contributions

XX and QL designed the research. ML, TL, XW, CY, TQ, and JL performed the experiments. MZ, ZL, and XC analyzed the data. ML, TL, and XW wrote the manuscript. All authors contributed to the article and approved the submitted version.
